# Mapping the Insecticide Resistance Landscape of Malaria Vectors in Odisha, India (1993–2024): A Scoping Review

**DOI:** 10.1155/jotm/8879127

**Published:** 2026-04-06

**Authors:** Tapan Kumar Barik, Poonam Sharma Velamuri, Kiran Bala Bhuyan, Sadai Sabar, Pruthiraj Mohapatra, Bijayalaxmi Sahu, Manoj Kumar Das, Sanghamitra Pati, Aditya Prasad Dash, Kamaraju Raghavendra

**Affiliations:** ^1^ Department of Zoology, Berhampur University, Bhanja, Bihar, 760007, Odisha, India, bamu.nic.in; ^2^ ICMR-Regional Medical Research Centre North East, Lahowal, Dibrugarh, 786010, Assam, India; ^3^ Department of Zoology, S. K. C. G. College, Paralakhemundi, Gajapati, 761200, Odisha, India; ^4^ ICMR–National Institute of Malaria Research, Field Unit, Ranchi, 834010, Jharkhand, India, nimr.org.in; ^5^ ICMR-Regional Medical Research Centre, Indian Council of Medical Research, Chandrasekharpur, Bhubaneswar, 751023, Odisha, India, icmr.nic.in; ^6^ ICMR-National Institute of Malaria Research, Sector-8 Dwarka, New Delhi, 110077, India, nimr.org.in

**Keywords:** Anopheles, insecticide resistance, insecticides, malaria vector, Odisha

## Abstract

**Background:**

Vector‐borne​ diseases (VBDs) are a serious threat to public health, globally. Different genera of mosquitoes, acting as disease vectors, transmit diseases such as malaria, filariasis, dengue, chikungunya, Zika fever, and others. Malaria in humans is caused by *Plasmodium* species transmitted by *Anopheles* mosquitoes. Odisha is one of the malaria‐endemic states in the country contributing to about 25% of the total (1.5–2.0 million) reported malaria cases and 30% deaths annually. Chemical insecticides and interventions have been effective in controlling vectors, and their continued usage resulted in widespread insecticide resistance in mosquito disease vectors, limiting their efficacy.

**Methods:**

Insecticide resistance data in malaria vector species in Odisha were compiled from the published peer‐reviewed literature and other validated sources by search using keywords Anopheles, anopheles species, insecticide, susceptibility, mortality, survival, insecticide resistance, Odisha, Orissa, etc. The inclusion and exclusion criteria were followed to ensure the selection of quality data published between 1993 and 2024. Data from 28 published sources were collated for compilation and adapted for this scoping review. In adult susceptibility tests, mortality rates were categorized as susceptible/resistant to the specific insecticide following the World Health Organization (WHO) criterion: susceptible: > 98% mortality; possible resistance: between 90% and 97% mortality; and confirmed resistance: < 90% mortality. Intensity bioassay data from 10 southern districts were presented for the major vector *Anopheles culicifacies*.

**Results:**

Odisha with 30 administrative districts has five defined ecotypes, coastal plains, central plateaus, central mountainous and highlands region, and western rolling and main flood plains. *An. culicifacies,* a major malaria vector, is reportedly prevalent in all 30 districts of Odisha, *An. fluviatilis* in 24 districts, *An. annularis* in 22, and *An*. *minimus* in two districts. *An. culicifacies* was the most resistant species to DDT (dichlorodiphenyltrichloroethane) (26 districts), malathion (17), and deltamethrin (10 districts). Limited data were available for other *Anopheles* species. Analyzed data on the status of resistance to different insecticides in anopheles vector species are provided.

**Conclusion:**

The emergence of multiple insecticide resistance in *An*. *culicifacies* signifies the urgency for effective management strategies. Insecticide resistance management requires regular monitoring and implementation of innovative approaches for vector control, emphasizing the need for insecticides with a novel mode of action and integrated vector management methods. Gaps in the data and ways to address them and issues on insecticide resistance management are discussed.

## 1. Introduction

Vector‐borne diseases (VBDs) arise from pathogens transmitted by arthropod vectors. Diseases such as dengue, Chagas disease, Japanese encephalitis, leishmaniasis, lymphatic filariasis (LF), malaria, and yellow fever pose substantial risks to over 80% of the World’s population in tropical and subtropical regions [1]. Among the VBDs, human malaria is prominent and is caused by the protozoan parasite, *Plasmodium* spp., transmitted by the mosquito, *Anopheles* spp. Globally 249 million malaria cases were estimated from 85 malaria‐endemic countries and areas (including the territory of French Guiana), a global increase of 5 million cases in 2023 compared to 2021 [2]. Odisha (earlier Orissa), an eastern state, is endemic among the states in India and has consistently been the major contributor toward morbidity and mortality, contributing to about 25% of the total (1.5–2.0 million) reported malaria cases and 30% deaths annually [3]. The state had reported 41,971 malaria cases in 2023, which is the highest number of malaria cases in 2023 among all the states [4].

Among the 58 species of anopheline mosquitoes documented in India, six are primary malaria vector species, namely, *Anopheles baimaii*, *An. culicifacies*, *An. fluviatilis*, *An. minimus*, *An. stephensi*, and *An. sundaicus,* and few secondary vectors, namely, *An. annularis*, *An. nivipes*, *An. philippinensis*, and *An. varuna,* with varied distributions across regions of India [5]. Of these six species, *An*. *culicifacies* is the predominant vector that breeds in stagnant clean waters, such as riverbed pools and puddles, and transmits malaria in rural plain areas (∼60–70% of new cases of malaria annually); *An*. *fluviatilis* breeds in slow‐moving waters and transmits malaria in forested areas and foothills (∼15% of cases); *An. stephensi* is the key vector for urban malaria transmission and in rural areas in parts of Rajasthan state (∼12% of cases) and is mainly a container breeder; and the remaining 8% cases were transmitted by three species, namely, *An*. *minimus s*.*s*, which breeds in streams within the foothills in the northeast and in some eastern states; *An*. *baimaii,* primarily an exophilic and exophagic mosquito, inhabits forested areas in the northeastern states and breeds in water receptacles such as animal hoof marks and puddles; *An*. *sundaicus* (cytotype D) breeds in brackish water, presently reported from Andaman and Nicobar Islands [5]. Except *An. stephensi*, all are species complexes and exhibit a diverse role in malaria transmission with associated complexity in control. In this scoping review, we presented data on *An*. *annularis* reported vector of local importance [5] prevalent in a few districts in Odisha.

Historically, the use of chemical insecticides has been effective for vector control and their continued use in public health in time and space led to the development of widespread insecticide resistance in mosquito disease vectors. Insecticides with a novel mode of action from other insecticide classes, namely neonicotinoids, pyrroles and others, are in testing, and evaluation phases and a few interventions are already recommended by WHO (World Health Organization) for use in vector control to manage insecticide resistance [5]. Insecticide resistance in *Anopheles* mosquitoes is a growing challenge for malaria control worldwide, and increasing pyrethroid resistance is documented globally. A study in Uganda revealed that widespread insecticide resistance in *An. gambiae* and *An*. *arabiensis* discounted the efficacy of insecticide‐treated nets (ITNs) [6]. The agricultural insecticide use may also have resulted in the development of insecticide resistance in mosquito [7]. Neonicotinoids with a novel mode of action also contributed to insecticide resistance in *An. coluzzii* [8]. A total of 78 countries registered confirmed resistance to at least one insecticide in one malaria vector species from one mosquito collection site during the period 2010–2020 [2].

Three classes of insecticides are in use in public health in India for adult vector control. For indoor residual sprays (IRSs), namely, organochlorine, organophosphate, and pyrerthroids pyrethroid‐impregnated long‐lasting insecticidal nets (LLINs) are used. It may be noted that the term LLINs have been abbreviated as LNs and recently these are being referred to as ITNs. The primary malaria vector, *An*. *culicifacies*, showed multiple insecticide resistance in many states of India. Studies were conducted across 15 states in India during 2017–2020, and districtwise susceptibility data were presented [9]; the primary malaria vector *An. culicifacies* showed resistance to DDT (50/50 districts including two districts of Northeastern India), malathion (27/44 districts), and deltamethrin (17/44 districts). This species was resistant to DDT alone in 19 districts, double resistant to DDT–malathion in 16 districts, double resistant to DDT–deltamethrin in 6 districts, and triple resistant to DDT–malathion–deltamethrin in 9 districts [9]. The development of insecticide resistance in malaria vectors retards the disease control. In Odisha, a state with a high malaria burden, the primary vector *An*. *culicifacies* developed multiple insecticide resistance. Studies conducted between 2014 and 2018 across several districts revealed resistance to DDT, malathion, and deltamethrin [10] and retard the effectiveness of the interventions [11]. Hence, for strategizing the vector control options, regular monitoring of insecticide resistance is important, which provides information about resistance profile for cross‐ and multiple insecticide resistance and suggests appropriate vector control strategies for resistance management.

The objective of this scoping review is to present comprehensive data on insecticide resistance in prevalent malaria vectors in Odisha to strategize the vector control interventions by the antimalarial program.

## 2. Methods

### 2.1. Literature Search

This scoping review provides information on the prevalence of malaria vector species in Odisha collected from peer‐reviewed published literature and other validated data from institutional annual reports. The systematic search was conducted by using keywords Anopheles, Anopheles species, insecticide, susceptibility, mortality, survival, insecticide resistance, Odisha, Orissa, etc. The data were compiled as a scoping review of the available datasets from the year 1993 to 2025. The district is the unit for the reported data. No formal protocol was registered for this scoping review, but the methodology adhered to the Preferred Reporting Items for Systematic Reviews and Meta‐Analyses Extension for Scoping Reviews (PRISMA‐ScR) guidelines [12]. Data were primarily sourced from peer‐reviewed publications, with the final search conducted on September 25, 2025. This scoping review included data generated following WHO protocols using WHO‐specified discriminating concentrations. Eligible studies were identified through title and abstract screening, followed by detailed data in the full text.

Inclusion and exclusion criteria were established to ensure the reliability and quality of the analyzed information. Studies conducted in districts or specific localities within Odisha and the articles published between 1993 and 2025 were included in this scoping review. To maintain accuracy, the names of locations were verified for spelling errors and alternative names, where applicable. Studies based on non‐Anopheles species, lacking specific location details, and missing critical components, such as mortality data, insecticide types, or sample sizes, were excluded. Additionally, studies that employed nonstandard methodologies, nonprescribed discriminatingconcentrations, or duplicated data from previous reports were omitted. Only peer‐reviewed and published data were considered to ensure the inclusion of scientifically validated findings. Data were extracted into Microsoft Excel spreadsheets for analyses.

Data were collated using a structured format, which included vector species, year and location (district) of study, insecticide tested, WHO discriminating concentrations, number of mosquitoes tested, mortality rates, resistance interpretation (susceptible, possible resistance, and confirmed resistance), and intensity bioassay results (5x and 10x concentrations of the 1x discriminating concentration). Mortality response to insecticide exposure served as the criterion for assessing susceptibility status.

**FIGURE 1:**
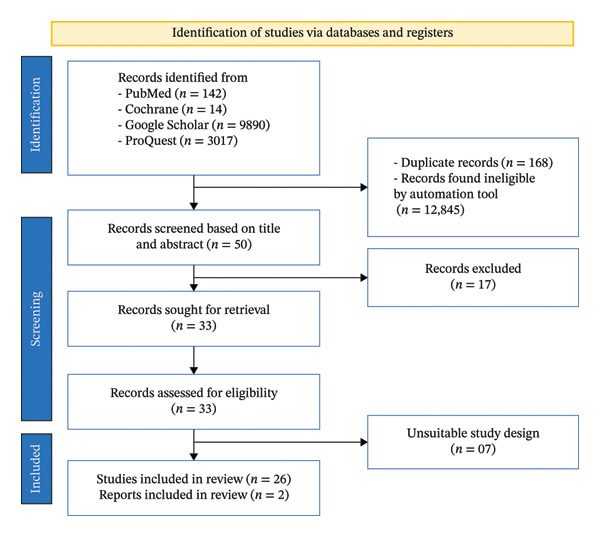
PRISMA flowchart of the selection process for the study.

A total of 13,063 records were available between 1993 and September 2025. After screening based on inclusion criteria, 28 studies were included in the final synthesis. A PRISMA flow diagram (Figure 1) illustrates the screening and selection process. The final dataset includes phenotypic insecticide susceptibility data for four malaria vector species, *An. culicifacies*, *An. fluviatilis*, *An. annularis*, and *An. minimus* across 26 of the 30 districts of Odisha. The data were presented by species and insecticide type, detailing the susceptibility status of vectors in each district. Key findings were summarized.

### 2.2. Susceptibility Test

The adult susceptibility data to insecticides in use for disease vector control were reportedly generated by the authors following standard WHO methods [13] using WHO‐impregnated papers with prescribed insecticide discriminating concentrations (DC), viz., DDT 4.0%, malathion 5.0%, deltamethrin 0.05%, and alphacypermethrin 0.05% (tentative DC). Wild‐collected/laboratory‐reared mosquitoes exposed generally in 4–5 replicates of 20–25 mosquitoes each (*n* = 100) and insecticide controls in two replicates of 25 mosquitoes each (*n* = 50). Data of some exposures with less numbers are indicated with hash in the footnote of the data tables. Exposures were generally made for one hour followed by the 24‐h holding period. Based on the recorded mortality rates in replicates, the population is designated as susceptible/resistant to the specific insecticide following the WHO criterion, susceptible: ≥ 98% mortality; possible resistance: between 90% and 97% mortality, and confirmed resistance: < 90% mortality [13–16].

### 2.3. Intensity Bioassay

Further to the above susceptibility tests, intensity bioassay data were available. These bioassays ascertain the intensity of resistance, i.e., “strength” of a resistance phenotype. Fd resistant to WHO‐prescribed discriminating concentration (DC 1x, i.e., 1 time is the WHO‐prescribed concentration, e.g., deltamethrin 0.05%) of the given insecticide (< 90% mortality) was exposed to higher concentrations of insecticides, i.e., 5 times (5x) and 10 times (10x) the prescribed discriminating concentration (1x) following the similar testing procedure described above for adult susceptibility tests. These tests facilitate assessing the operational usability of the insecticide being used in the intervention [13, 16]. The results of intensity bioassays are interpreted as “(i) mortality in the range of 98%–100% at 5x concentration indicates a low intensity resistance and further testing at 10x concentration is not necessary—*suggesting to continue the existing insecticide for vector control intervention;* (ii) mortality of < 98% at 5x concentration indicates a moderate resistance intensity—it is recommended to assay further at the 10x concentration; and (iii) mortality between 98% and 100% at 10x concentration confirms a moderate resistance intensity. Mortality of < 98% at 10x concentration indicates a high resistance intensity” [13–16]. Notably, if high resistance is evidenced in the intensity bioassays in exposures to 10x concentration, operational failure is likely and a change of insecticide can be preferred.

## 3. Results

### 3.1. Malaria Vectors and Their Distribution in Odisha

Odisha, with its 30 administrative districts is characterized by five distinct ecotypes: coastal plains, central plateaus, central mountainous and highland regions, western rolling regions, and main flood plains. Malaria transmission varies significantly across these ecotypes due to differences in ecological and climatic factors. The primary malaria vector *An*. *culicifacies* is prevalent in all the districts [9, 17–32], while other vectors, namely, *An. fluviatilis*, *An. annularis*, *An. minimus,* and *An. sundaicus*, are distributed based on the ecological attributes. This vector distribution informs the transmission dynamics of malaria and supports the development of targeted, species‐specific control strategies.

*An. fluviatilis* was reported in 24 districts [9, 18, 20, 21, 23, 26, 28–34], predominantly in the northern districts (Angul, Sundargarh), central (Cuttack, Mayurbhanj), and southern regions (Kalahandi, Koraput) but were reportedly not prevalent in districts Sambalpur and Kendrapara. *An. annularis* was found in 22 districts [19–21, 23–26, 29, 30, 35–37], including Angul, Sundargarh, and Rayagada. *An. minimus* inhabits four districts [36, 38, 39], mainly in the northern zone. The other important vector species *An. sundaicus*, though reported to be prevalent in two coastal districts, Ganjam and Puri six decades ago, however, were not found in the later surveys [40, 41].

**FIGURE 2:**
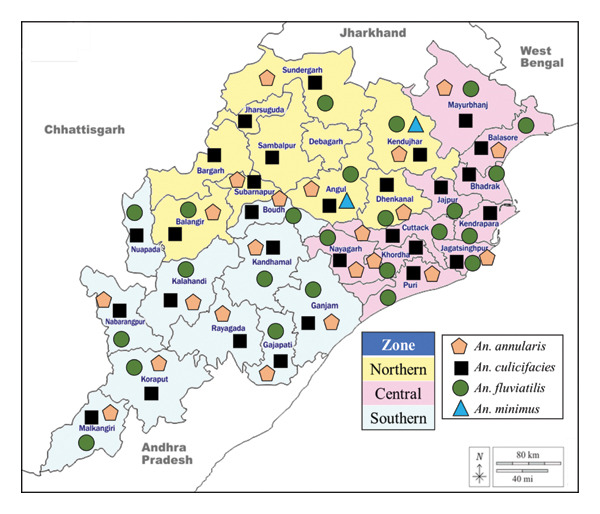
Distribution of malaria vectors of Odisha, India.

Regions with forested and hilly terrains, particularly in the southern, western, and northern districts (e.g., Koraput, Balangir, and Kalahandi), are at a higher risk of malaria due to their tropical climate, characterized by high temperatures, humidity, and rainfall, which create optimal breeding conditions for vectors and parasite development. Conversely, the eight coastal districts report low malaria incidence, except for localized hotspots probably due to the nonavailability of congenial conditions for breeding affecting the prevalence of malaria vectors in this region and subsequent disease transmission. Understanding these distribution patterns aids in designing effective vector management strategies tailored to regional ecological conditions (Figure 2 and Table 1).

**TABLE 1:** Distribution of malaria vectors in different districts in the three zones of Odisha, India.

Zone	*An. culicifacies* [Reference]	*An. fluviatilis* [Reference]	*An. annularis* [Reference]	*An. minimus* [Reference]
Northern	Angul [17]	Angul [20]	Angul [20, 35, 36]	Angul [36]
	Balangir [18]	Balangir [28]	Balangir [35]	
	Bargarh [19]			
	Deogarh/Debagarh—part of undivided Sambalpur district (data not available for this district)			
	Dhenkanal [20, 21]	Dhenkanal [20, 21]	Dhenkanal [20, 21]	
	Jharsuguda [22]			
	Kendujhar [18, 20, 22, 23]	Kendujhar [18, 20, 23]	Kendujhar [20, 23, 35, 37]	Kendujhar [36, 38, 39]
	Sambalpur [19]			
	Subarnapur [22]		Subarnapur [35]	
	Sundargarh [18, 24]	Sundargarh [18, 32]	Sundargarh [24]	

Central	Balasore [25]	Balasore [9, 18]	Balasore [25]	
	Bhadrak [9]	Bhadrak [9]		
	Cuttack [22, 26]	Cuttack [9]^,^	Cuttack [26]	
	Jagatsinghpur [20, 22]	Jagatsinghpur [9, 18]	Jagatsinghpur [20]	
	Jajpur [9]	Jajpur [9]		
	Kendrapara [9]	Kendrapara [9]		
	Khordha [20, 22]	Khordha [9, 20]	Khordha [20]	
	Mayurbhanj [18, 22]	Mayurbhanj [9, 18]	Mayurbhanj [35]	
	Nayagarh [20]	Nayagarh [20]	Nayagarh [20]	
	Puri [20]	Puri [9]	Puri [20]	

Southern	Boudh [27]	Boudh [33]	Boudh [35]	
	Gajapati [22]	Gajapati [28]	Gajapati [19, 35]	
	Ganjam [20, 22, 27]	Ganjam [20, 28]	Ganjam [20]	
	Kalahandi [18, 19]	Kalahandi [18, 26]	Kalahandi [26, 35]	
	Kandhamal [18, 28]	Kandhamal [18, 28]	Kandhamal [35]	
	Koraput [29, 30]	Koraput [29, 30, 34]	Koraput [29, 30]	
	Malkangiri [30–32]	Malkangiri [28, 30, 31]	Malkangiri [30, 35]	
	Nabarangpur [19]	Nabarangpur [28]	Nabarangpur [19, 35]	
	Nuapada [18, 22]	Nuapada [28]		
	Rayagada [18, 28]		Rayagada [19, 35]	

### 3.2. Vector Control in Odisha

Vector control in Odisha is reliant on chemical insecticides. Indoor residual spraying with DDT or synthetic pyrethroids (deltamethrin) has been carried out as the major vector control measure. In addition, deltamethrin LLINs have been distributed in 10 southern districts, namely, Balangir, Kalahandi, Kandhamal, Gajapati, Ganjam, Malkangiri, Nabarangpur, Nuapada, Rayagada, and Koraput [42]. These districts received LLINs (deltamethrin/alphacypermethrin LNs) from the Global Fund to fight AIDS, Tuberculosis and Malaria (GFATM) since 2017. The DDT‐IRS was withdrawn from ∼ 80% areas of study districts since 2017, and the last round was sprayed on September–October 2018.

### 3.3. Insecticide Susceptibility Status in Different Species

**FIGURE 3:**
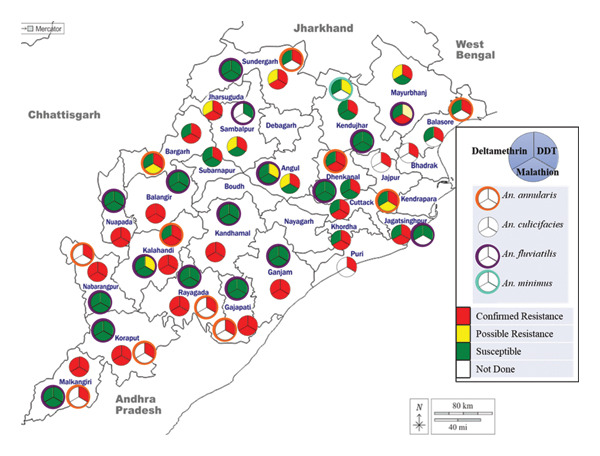
Insecticide susceptibility status of malaria vector species in Odisha, India.

Specieswise data on insecticide susceptibility are given in Tables 2, 3, and 4 and Figure 3. Specieswise susceptibility data are given in brief below.

**TABLE 2:** Insecticide susceptibility status of *An. culicifacies* to DDT, malathion, and deltamethrin in different districts of Odisha.

Sl. no	District (place)	Year	DDT (4%)			Malathion (5%)			Deltamethrin (0.05%)			Reference
			No. exposed (*n*)	Mortality (%)	Status	No. exposed (*n*)	Mortality (%)	Status	No. exposed (*n*)	Mortality (%)	Status	
1	Angul	2020	120	28	CR	120	98	S	120	95.2	PR	[9]
		2009	80^#^	9.7	CR	40^#^	100	S	30^#^	96.3	PR	[19, 22]

2	Balangir	2018	≥ 100	28	CR	≥ 100	74	CR	≥ 100	82	CR	[10]
		2017	≥ 100	37	CR	≥ 100	62	CR	≥ 100	84	CR	
		2010	106	12.3	CR	105	80	CR	104	94.2	PR	[19, 28]
		2009	502	7.8	CR	511	74.4	CR	494	96	PR	[19, 22]
		2002	60^#^	23.3	CR	60^#^	68.3	CR	60^#^	95	PR	[18, 19]

3	Balasore	2010	NA	NA	CR	ND	ND	ND	NA	NA	S	[25]

4	Bargarh	2015–2017	NA	17.2	CR	NA	72	CR	NA	94.3	PR	[43]
		2009	300	12.5	CR	280	72.3	CR	340	98.8	S	[19], [22]

5	Bhadrak	2017‐2018	30^#^	50	CR	ND	ND	ND	ND	ND	ND	[9]

6	Cuttack	2017‐2018	30^#^	60	CR	ND	ND	ND	ND	ND	ND	
		2015–2017	NA	19	CR	NA	70	CR	NA	98.5	S	[43]
		2009	100	20	CR	74^#^	90	CR	90^#^	100	S	[19, 22]

7	Dhenkanal	2009	30^#^	9.3	CR	20^#^	100	S	20^#^	100	S	[19, 22]
		2008–2010	10–15^#^	23–40	CR	7–15^#^	25–50	CR	3–12^#^	100	S	[21]

8	Gajapati	2018	≥ 100	20	CR	≥ 100	72	CR	≥ 100	82	CR	[10]
		2017	≥ 100	24	CR	≥ 100	46	CR	≥ 100	80	CR	
		2010	103	15.5	CR	105	83.8	CR	105	82.9	CR	[19, 28]
		2009	300	12.6	CR	230	70.3	CR	280	98	S	[19, 22]
	(Guma)	2005	15^#^	20	CR	ND	ND	ND	15^#^	100	S	[19]
	(Mohana)		15^#^	26.6	CR	ND	ND	ND	15^#^	100	S	

9	Ganjam	2018	≥ 100	20	CR	≥ 100	71	CR	≥ 100	82	CR	[10]
		2017	≥ 100	14	CR	≥ 100	68	CR	≥ 100	85	CR	
		2010	102	14.7	CR	101	70.3	CR	104	95.2	PR	[19, 28]
		2009	30^#^	18.4	CR	20^#^	85	CR	30^#^	100	S	[19, 22]

10	Jagatsinghpur	2017‐2018	30^#^	46.6	CR	ND	ND	ND	ND	ND	ND	[9]
		2009	100	23	CR	80^#^	85.5	CR	90^#^	100	S	[19, 22]

11	Jajpur	2017‐2018	30^#^	60	CR	ND	ND	ND	ND	ND	ND	[9]

12	Jharsuguda	2022	60^#^	25	CR	ND	ND	ND	60^#^	95	PR	[44]
		2009	260	12.6	CR	240	40	CR	240	96.7	PR	[19, 22]

13	Kalahandi	2020	120	33	CR	120	71	CR	120	92.5	PR	[9]
		2018	≥ 100	19	CR	≥ 100	66	CR	≥ 100	86	CR	[10]
		2017	≥ 100	23	CR	≥ 100	50	CR	≥ 100	77	CR	
			ND	ND	ND	ND	ND	ND	75^#^	74.6	CR	[32]
		2015–2017	NA	9	CR	NA	62	CR	NA	81.3	CR	[43]
		2014	105	12.4	CR	111	60.4	CR	131	79.4	CR	[19, 45]
		2010	105	14.3	CR	105	86.7	CR	104	81.7	CR	[19, 28]
		2009	76^#^	11.8	CR	120	78.3	CR	120	81.6	CR	[19, 22]
		2002	60^#^	12	CR	60^#^	88.3	CR	60^#^	96.7	PR	[19]

14	Kandhamal	2018	≥ 100	10	CR	≥ 100	76	CR	≥ 100	80	CR	[10]
		2017	≥ 100	19	CR	≥ 100	53	CR	≥ 100	81	CR	
		2010	105	9.5	CR	109	77.6	CR	109	96.3	PR	[19, 28]
	(Phulbani)	2009	93^#^	6.4	CR	98^#^	59.1	CR	96^#^	93.7	PR	[19, 22]
	(Phulbani)	2002	60^#^	20	CR	60^#^	100	S	60^#^	100	S	[18, 19]

15	Kendujhar	2009	40^#^	11.1	CR	30^#^	100	S	20^#^	100	S	[19, 22]
		2002	50^#^	14	CR	ND	ND	ND	80^#^	100	S	[18, 19]

16	Khordha	2017‐2018	30^#^	56.6	CR	ND	ND	ND	ND	ND	ND	[9]
		2009	20^#^	20	CR	20^#^	80	CR	30^#^	100	S	[19, 22]

17	Koraput	2019	NA	12–13	CR	ND	ND	ND	ND	ND	ND	[46]
		2018	≥ 100	17	CR	≥ 100	69	CR	≥ 100	78	CR	[10]
		2017	≥ 100	14	CR	≥ 100	66	CR	≥ 100	83	CR	
			ND	ND	ND	ND	ND	ND	75^#^	73.3	CR	[32]
		2014	111	15.3	CR	111	66.7	CR	112	76.8	CR	[19, 45]
		2013‐2014	NA	9.8	CR	ND	ND	ND	NA	76.1	CR	[47]
		2010	111	13.5	CR	111	76.6	CR	123	98.4	S	[19, 28]
			753	8.1	CR	ND	ND	ND	ND	ND	ND	[30]
		2002	33^#^	21.2	CR	ND	ND	ND	ND	ND	ND	[19, 48]

18	Malkangiri	2018	≥ 100	14	CR	≥ 100	78	CR	≥ 100	76	CR	[10]
		2017	≥ 100	11	CR	≥ 100	64	CR	≥ 100	80	CR	
			ND	ND	ND	ND	ND	ND	75^#^	84	CR	[32]
		2015	90^#^	53.3	CR	ND	ND	ND	ND	ND	ND	[49]
		2014	111	12.6	CR	105	76.2	CR	119	84	CR	[19, 45]
		2010	105	15.2	CR	110	75.5	CR	109	86.2	CR	[19, 28]
			527	12.1	CR	ND	ND	ND	ND	ND	ND	[30]
		2002	90^#^	21.1	CR	102	35.3	CR	51^#^	100	S	[19, 48]
		1993	925	0–10	CR	ND	ND	ND	ND	ND	ND	[19, 31]

19	Mayurbhanj	2017‐2018	30^#^	40	CR	ND	ND	ND	ND	ND	ND	[9]
		2009	30^#^	14.8	CR	20^#^	100	S	27^#^	96.3	PR	[19, 22]
	(Badampahar, Rangamatia)	2005	15^#^	20	CR	ND	ND	ND	15^#^	100	S	[19]
		2002	40^#^	62.5	CR	40^#^	50	CR	60^#^	100	S	[18, 19]

20	Nabarangpur	2018	≥ 100	21	CR	≥ 100	78	CR	≥ 100	82	CR	[10]
		2017	≥ 100	17	CR	≥ 100	61	CR	≥ 100	83	CR	
			ND	ND	ND	ND	ND	ND	75^#^	85.3	CR	[32]
		2014	105	11.4	CR	110	70.9	CR	113	72.6	CR	[19, 45]
		2010	109	13.8	CR	126	63.5	CR	114	96.5	PR	[19, 28]
	(Nandahandi)	2005	15^#^	20	CR	ND	ND	ND	15^#^	100	S	[19]
	(Papadahandi)		15^#^	26.6	CR	ND	ND	ND	15^#^	100	S	
	(Tentulikhunti)		15^#^	20	CR	ND	ND	ND	15^#^	100	S	

21	Nuapada	2018	≥ 100	14	CR	≥ 100	83	CR	≥ 100	80	CR	[10]
		2017	≥ 100	27	CR	≥ 100	62	CR	≥ 100	85	CR	
		2010	107	15	CR	98^#^	67.3	CR	89^#^	100	S	[19, 28]
		2009	60^#^	3.3	CR	49^#^	93.8	PR	59^#^	88.1	CR	[19, 22]
		2002	60^#^	8.3	CR	60^#^	75	CR	60^#^	81.7	CR	[18, 19]

22	Puri	2017‐2018	30^#^	60	CR	ND	ND	ND	ND	ND	ND	[9]

23	Rayagada	2018	≥ 100	17	CR	≥ 100	66	CR	≥ 100	81	CR	[10]
		2017	≥ 100	16	CR	≥ 100	59	CR	≥ 100	84	CR	
			ND	ND	ND	ND	ND	ND	75^#^	80	CR	[32]
		2014	135	12.6	CR	130	63.1	CR	131	81.7	CR	[19, 45]
		2010	102	16.7	CR	105	77.6	CR	108	89.8	CR	[19, 28]
		2009	272	23.1	CR	278	90.6	PR	270	89.2	CR	[19, 22]
	(Bissam Cuttack)	2005	15^#^	26.6	CR	ND	ND	ND	15^#^	100	S	[19]
			15^#^	13.3	CR	ND	ND	ND	15^#^	100	S	
	(Muniguda)	2002	60^#^	15	CR	60^#^	100	S	60^#^	100	S	[18, 19]

24	Sambalpur	2009	30^#^	14.8	CR	20^#^	100	S	27^#^	96.3	PR	[19, 22]

25	Subarnapur	2009	30^#^	9.3	CR	20^#^	100	S	20^#^	100	S	[19, 22]

26	Sundargarh	2013	NA	29	CR	NA	70.7	CR	NA	100	S	[50]
		2009	280	25.9	CR	260	70.7	CR	260	95.1	PR	[19, 22]
		2008	NA	NA	CR	NA	NA	S	ND	ND	ND	[51]
		2005	NA	15	CR	NA	100	S	NA	100	S	[52]
		2002	100	12	CR	100	100	S	100	100	S	[18, 19]
			NA	16.4	CR	NA	100	S	NA	100	S	[53]

*Note:* NA: data not available, ND: test not done, No. Exposed (n): number of mosquitoes exposed; status: S: susceptible, ≥ 98% mortality; PR: possible resistance, 90%–97% mortality; CR: confirmed resistance, < 90% mortality [13–16].

^#^where *n* < 100.

**TABLE 3:** Insecticide susceptibility status of *An. fluviatilis* to DDT, malathion, and deltamethrin in different districts of Odisha.

Sl. no	District	Year	DDT (4%)			Malathion (5%)			Deltamethrin (0.05%)			Reference
			No. exposed (*n*)	Mortality (%)	Status	No. exposed (*n*)	Mortality (%)	Status	No. exposed (*n*)	Mortality (%)	Status	
1	Angul	2019	100	91	PR	100	100	S	100	100	S	[9]
		2009	8^#^	100	S	ND	ND	ND	ND	ND	ND	[19]

2	Balangir	2010	56^#^	100	S	54^#^	100	S	54^#^	100	S	[19, 28]

3	Dhenkanal	2008–2010	30^#^	100	S	7^#^	100	S	8^#^	100	S	[21]

4	Ganjam	2010	50^#^	100	S	52^#^	100	S	52^#^	100	S	[19, 28]

5	Gajapati	2010	62^#^	100	S	61^#^	100	S	57^#^	100	S	[19, 28]

6	Jharsuguda	2022	60^#^	98.3	S	ND	ND	ND	60^#^	100	S	[44, 54]

7	Kalahandi	2019	100	90	PR	100	98	S	100	99	S	[9]
		2010	79^#^	100	S	62^#^	100	S	79^#^	100	S	[19, 28]
		2002	60^#^	100	S	60^#^	100	S	60^#^	100	S	[18, 19]

8	Kandhamal	2010	60^#^	100	S	64^#^	100	S	63^#^	100	S	[19, 28]
	(Phulbani)	2002	60^#^	100	S	40^#^	100	S	40^#^	100	S	[18, 19]

9	Kendujhar (Banspal)	2009	6^#^	100	S	ND	ND	ND	ND	ND	ND	[19]
		2009	52^#^	100	S	ND	ND	ND	52^#^	100	S	[19, 55]
		2002	100	100	S	40^#^	100	S	120	100	S	[18, 19]

10	Koraput	2013‐2014	NA	100	S	ND	ND	ND	ND	ND	ND	[47]
		2010	55^#^	100	S	66^#^	100	S	55^#^	100	S	[19, 28]
			90^#^	100	S	ND	ND	ND	ND	ND	ND	[30]
		2002	557	100	S	210	100	S	290	100	S	[19, 48]

11	Malkangiri	2010	67^#^	100	S	44^#^	100	S	56^#^	100	S	[19, 28]
			110	100	S	ND	ND	ND	ND	ND	ND	[30]
		2002	493	100	S	192	100	S	108	100	S	[19, 48]
		1993	260	100	S	ND	ND	ND	ND	ND	ND	[19, 31]

12	Mayurbhanj	2009	20^#^	100	S	ND	ND	ND	ND	ND	ND	[19]
		2002	40^#^	95	PR	40^#^	87.5	CR	40^#^	100	S	[18, 19]

13	Nabarangpur	2010	32^#^	100	S	20^#^	100	S	24^#^	100	S	[19, 28]

14	Nuapada	2010	54^#^	100	S	54^#^	100	S	58^#^	100	S	[19, 28]

15	Rayagada	2013	100	100	S	ND	ND	ND	100	100	S	[19, 56]
		2010	60^#^	100	S	57^#^	100	S	58^#^	100	S	[19, 28]

16	Sambalpur	2009	20^#^	100	S	ND	ND	ND	ND	ND	ND	[19]

17	Sundargarh	2008	NA	NA	S	NA	NA	S	ND	ND	ND	[51]
		2005	NA	100	S	NA	100	S	NA	100	S	[52]
		2002	NA	100	S	NA	100	S	NA	100	S	[53]
		2002	100	100	S	60^#^	100	S	100	100	S	[18, 19]
		2001	NA	100	S	NA	100	S	NA	100	S	[19, 57]
		2000	NA	100	S	ND	ND	ND	NA	100	S	[19, 57]

*Note:* NA: data not available, ND: test not done, No. exposed (n); number of mosquitoes exposed; status: S: susceptible, ≥ 98% mortality; PR: possible resistance, 90%–97% mortality; CR: confirmed resistance, < 90% mortality [13–16].

^#^where *n* < 100.

**TABLE 4:** Insecticide susceptibility status of other anophelines to DDT, malathion, and deltamethrin in different districts of Odisha.

Sl. no	Species	District (place)	Year	DDT (4%)			Malathion (5%)			Deltamethrin (0.05%)			Reference
				No. exposed (*n*)	Mortality (%)	Status	No. exposed (*n*)	Mortality (%)	Status	No. exposed (*n*)	Mortality (%)	Status	
1	*An. annularis*	Balasore	2010	NA	NA	CR	ND	ND	ND	NA	NA	S	[25]
		Bargarh	2015–2017	NA	36	CR	NA	94.5	PR	NA	98	S	[43]
		Cuttack		NA	34.5	CR	NA	95.2	PR	NA	99	S	
		Dhenkanal	2010–2013	NA	30–45	CR	NA	30–45	CR	NA	100	S	[33]
			2008–2010	30^#^	30	CR	20^#^	45	CR	20^#^	100	S	[21]
		Gajapati (Gumma)	2005	15^#^	13.3	CR	ND	ND	ND	ND	ND	ND	[19]
		Kalahandi	2015–2017	NA	18.2	CR	NA	76.6	CR	NA	94	PR	[43]
		Koraput	2010	308	18.7	CR	ND	ND	ND	ND	ND	ND	[30]
		Malkangiri		220	25.4	CR	ND	ND	ND	ND	ND	ND	
		Nabarangpur (Papadahandi)	2005	15^#^	13.3	CR	ND	ND	ND	ND	ND	ND	[19]
		Rayagada (Muniguda)		15^#^	20	CR	ND	ND	ND	ND	ND	ND	
		Sundargarh	2000	NA	5.8	CR	ND	ND	ND	NA	100	S	[19, 57]

2	*An. minimus*	Kendujhar (Banspal)	2009	52^#^	96.2	PR	ND	ND	ND	52^#^	100	S	[19, 55]
		Kendujhar	2003	21^#^	86	CR	ND	ND	ND	45^#^	100	S	[19, 38]

*Note:* NA: data not available, ND: test not done, No. exposed (n); number of mosquitoes exposed; status: S: susceptible, ≥ 98% mortality; PR: possible resistance, 90%–97% mortality; CR: confirmed resistance, < 90% mortality [13–16].

^#^where *n* < 100.

*An. culicifacies*: In various studies during the reviewed period, it was observed that this species exhibited confirmed resistance in all 26 districts against DDT (data for districts Boudh, Deogarh, Kendrapara, and Nayagarh were not available). The species was reported to be resistant to malathion in 17 districts—Balangir, Bargarh, Cuttack, Dhenkanal, Gajapati, Ganjam, Jagatsinghpur, Jharsuguda, Kalahandi, Kandhamal, Khordha, Koraput, Malkangiri, Nabarangpur, Nuapada, Rayagada, and Sundargarh—and susceptible in five districts (Angul, Kendujhar, Mayurbhanj, Sambalpur, Subarnapur), and data were not available in four districts (Balasore. Bhadrak, Jajpur, and Puri). *An. culicifacies* reported confirmed resistance to deltamethrin in 10 districts (Balangir, Gajapati, Ganjam, Kalahandi, Kandhamal, Koraput, Malkangiri, Nabarangpur, Nuapada, and Rayagada), possible resistance in six districts, namely, Angul, Bargarh, Jharsuguda, Mayurbhanj, Sambalpur, and Sundargarh, and susceptibility in seven districts, namely, Balasore, Cuttack, Dhenkanal, Jagatsinghpur, Kendujhar, Khordha, and Subarnapur, and data were not available from three districts, namely, Bhadrak, Jajpur, and Puri. It was observed from the datasets that there were extensive studies in many districts and multiple studies on this species in different years.

*An. fluviatilis*: Susceptibility data from 17 districts were available. To DDT, this species was reported susceptible in 14 districts excluding Angul, Kalahandi, and Mayurbhanj districts, where it showed possible resistance. Of the 17 districts, this species reported susceptibility to malathion in 14 districts and resistance in district Mayurbhanj, and data were not available for districts Sambalpur and Jharsuguda. *An. fluviatilis* was reported susceptible to deltamethrin in 16 districts, and data were not available from Sambalpur district. *An*. *fluviatilis* displayed susceptibility to malathion, cyfluthrin, and deltamethrin in the two tribal districts (Angul and Kalahandi), with mortality rates ranging from 98% to 100% [9]. In four districts, resistance data from two locations were noted, while in five districts (Kalahandi, Kendujhar, Koraput, Malkangiri, and Sundargarh), three or more locations were found, indicating more comprehensive coverage. However, the insecticide resistance status of *An*. *fluviatilis* from the remaining 13 districts was not available.

*An. annularis*: This species was reported resistant to DDT in the surveyed 10 districts (Bargarh, Cuttack, Dhenkanal, Gajapati, Kalahandi, Koraput, Malkangiri, Nabarangpur, Rayagada, and Sundargarh) and confirmed resistance to malathion in two districts (Dhenkanal and Kalahandi) and possible resistance in Bargarh and Cuttack, while data were not available for malathion in the other six districts. However, the species was susceptible to deltamethrin in Balasore, Bargarh, Cuttack, Dhenkanal, and Sundargarh districts, and showed possible resistance in Kalahandi, while data were not available in the remaining four districts.

*An. minimus*: Data were available only from Kendujhar district where it has shown possible resistance to DDT and was susceptible to deltamethrin, while susceptibility data to malathion were not available.

### 3.4. Resistance Status to Different Insecticides

The major vector species *An. culicifacies* was reportedly widespread resistant. *An. culicifacies* is found to possess double resistance to DDT–deltamethrin and DDT–malathion and triple resistance to DDT–malathion–deltamethrin. While other vector species have shown varied resistance among the districts and not as widespread as *An. culicifacie*s. *An. fluviatilis,* also registered double resistance to DDT–malathion in Mayurbhanj. Other vector species have registered resistance to DDT alone or sporadic reports that are not of immediate operational relevance. The reported insecticide‐ and specieswise data were analyzed districtwise and are as follows:

#### 3.4.1. Resistance to DDT

*An. culicifacies*exhibited resistance to DDT in all 26 districts across the districts of Odisha and to other insecticides as well. Similarly, *An*. *fluviatilis* in three districts (Angul, Kalahandi, and Mayurbhanj) showed possible resistance and *An*. *annularis* showed resistance to DDT in nine districts (Balasore, Bargarh, Cuttack, Kalahandi, Koraput, Malkangiri, Nabarangpur, Rayagada, and Sundargarh), while *An*. *minimus* showed possible resistance to DDT in Kendujhar district. These findings underscore the widespread prevalence of DDT resistance among *Anopheles* mosquito species across different districts of Odisha.

#### 3.4.2. Resistance to Malathion

*An. culicifacies* exhibited confirmed resistance to malathion in all 17 districts of Odisha, while *An*. *fluviatilis* was reported to be resistant in one district (Mayurbhanj), and *An. annularis* showed confirmed resistance in Dhenkanal and Kalahandi and possible resistance in Bargarh and Cuttack districts.

#### 3.4.3. Resistance to Deltamethrin

*An. culicifacies* showed confirmed resistance to deltamethrin in 10 districts (Balangir, Gajapati, Ganjam, Kalahandi, Kandhamal, Koraput, Malkangiri, Nabarangpur, Nuapada, and Rayagada) and possible resistance in Angul, Bargarh, Jharsuguda, Mayurbhanj, Sambalpur, and Sundargarh, and *An. annularis* showed possible resistance in Kalahandi, while. *An. fluviatilis* was susceptible in all the surveyed districts.

#### 3.4.4. Double Resistance to DDT and Malathion

*An. culicifacies* exhibited double resistance to DDT and malathion in four districts (Cuttack, Dhenkanal, Jagatsinghpur, and Khordha). Notably, *An. fluviatilis* did not report double resistance, while *An. annularis* reported double resistance to DDT and malathion in two districts (Dhenkanal and Kalahandi).

#### 3.4.5. Double Resistance to DDT and Deltamethrin

*An. culicifacies* alone demonstrated double resistance to both DDT and deltamethrin in the three districts (Angul, Mayurbhanj, and Sambalpur).

#### 3.4.6. Triple Resistance to DDT, Malathion, and Deltamethrin

*An. culicifacies* alone exhibited triple resistance to insecticides in 10 districts (Balangir, Gajapati, Ganjam, Kalahandi, Kandhamal, Koraput, Malkangiri, Nabarangpur, Nuapada, and Rayagada districts) of Odisha. This type of resistance is a significant challenge as it reduces the effectiveness of insecticide‐based interventions.

### 3.5. Intensity Bioassays

**FIGURE 4:**
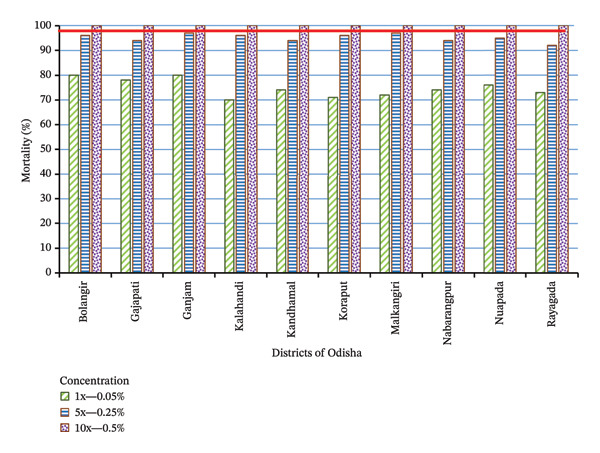
Resistance intensity bioassay in *An. culicifacies* to 0.05% (1x), 0.25% (5x), and 0.5% (10x) concentrations of deltamethrin in 10 southern districts of Odisha, India [42]. Resistance intensity criterion status based on % mortality at different discriminating concentrations—low intensity: DC5x ≥ 98% mortality; moderate intensity: DC5x < 98% mortality and DC10x ≥ 98% mortality; and high intensity: DC10x < 98% mortality [13, 16].

Bioassays for assessing the intensity of resistance were conducted on deltamethrin‐resistant *An. culicifacies* in 10 southern districts (Balangir, Gajapati, Ganjam, Kalahandi, Kandhamal, Koraput, Malkangiri, Nabarangpur, Nuapada, and Rayagada) from June to September, 2019. *An. culicifacies* showed confirmed resistance to 1x DC of deltamethrin (0.05%) in all 10 districts with mortality < 90% and 92% to 97% against 5x DC of deltamethrin (0.25%), indicating moderate resistance, but reported 100% mortality against 10x DC of deltamethrin (0.50%) that confirmed the absence of high resistance (Figure 4). As per the WHO criterion, this result indicated that operational failure of vector control was unlikely with the use of deltamethrin/alphacypermethrin‐LNs. Thus, the use of synthetic pyrethroid‐LNs for malaria vector control in these districts can be continued.

## 4. Discussion

For compiling insecticide resistance data, 1993 was taken as the baseline year. Available records from 1993 to September 2025 were retrieved using appropriate keywords from various sources. It was observed that data retrieved prior to 1993 were nil in our searches. Thus, the earliest insecticide resistance dataset identified in our search was 1993, similar to in an earlier review for years 1991–2016 [19]. Following 1993, the next dataset appeared only in 2000, indicating a clear gap in the data availability. Subsequent years also showed sporadic reporting. The earlier review [19] included 13 records from the state Odisha, whereas the present scoping review could compile data from 28 records that included a recent multistate study [9]. Thus, the current review includes 14 additional records, which was double the number of records available in the earlier review [19]. From the 28 records considered for the present review, 13 were for prevalence of vectors and insecticide resistance data in different districts of Odisha, while 15 were exclusively for insecticide resistance data.

This study discusses the available data on insecticide susceptibility/resistance in primary and secondary vectors of malaria in Odisha. Major malaria vector *An. culicifacies* is reportedly prevalent in 29 of 30 districts of Odisha (data of one district, Deogarh, was not available), *An. fluviatilis* in 25 districts, *An. annularis* in 22 districts, and *An. minimus* in 4 districts. Data were abundant for *An. culicifacies*, owing to its wide distribution.

The data presented in this article provide the detailed susceptibility status of malaria vectors in Odisha to different insecticides used for IRS in India and also provide information on resistance to single insecticides (DDT/malathion/deltamethrin), double resistance (DDT + malathion/DDT + deltamethrin), and triple resistance (DDT + malathion + deltamethrin). The data were not available systematically in time and space, and most of the data obtained were for *An. culicifacies* as this is the most prevalent species. A similar trend in testing was observed in an earlier review by Raghavendra et al. [19]. The collated data in the present scoping review included data from 26 of the 30 districts (data were not available from four districts, Deogarh, Boudh, Kendrapara, and Nayagarh). For other vector species, the reported data were less, which depended on the prevalence of different species specific to some geographies in the state and resultant number of tests conducted. Resistance in *An. culicifacies* to DDT was reported in all 26 districts, to malathion in 17 districts, and to deltamethrin in 10 districts; double insecticide resistance to DDT and malathion was reported in four districts and DDT and deltamethrin in three districts, and triple insecticide resistance to DDT, malathion, and deltamethrin was reported from 10 surveyed districts. The vector management of *An. culicifacies* is important for disease control as this species epitomizes the burden of insecticide resistance in malaria vectors. Similarly, an earlier review by Raghavendra et al. [19] reported DDT‐resistance in *An. culicifacies* in all 20 districts, while to malathion, it was in 15 of the 20 districts and to deltamethrin in 5 of the 20 districts. Similarly, in the recent data published by Raghavendra et al. [9], complete data were available for DDT from nine districts, which reported resistance. The data on DDT resistance in this species was 100% in all the districts in the three datasets in 2017 [19] (review), in 2022 [9] (study), and in this present review. Comparing the reviews of 2017 and the present review, there is a small increase in malathion resistance from 75% of the districts of the total reported districts in 2017 to 77%, while to deltamethrin, it increased from 25% districts in 2017 review to 43% in the present scoping review. The observed increase in the proportion of districts showing resistance to malathion and deltamethrin was not more, but the trend indicates that it may further increase with continued use of insecticides.

The precipitation of resistance is a dynamic process of evolution, and it is dependent on various factors, viz., genetic, biological, and population attributes, and mainly dependent on insecticide selection pressure on the population in time and space. The concept of “tipping point” [58] describes that initially the resistance gene frequency in the population may be significantly low (10^−6^) and may take a long time to reach a detectable level (1%), and if the population reaches the tipping point at a given frequency, the population may take few more generations to increase the resistance gene frequency. The best option for the management of resistance in field is to avoid or delay the onset of resistance. For both the options, rational use of insecticides is important. Furthermore, once the resistance gene stabilizes in the population, attempts to reverse the resistance may prove futile. A study in Surat, Gujarat state in India, on the stability of insecticide resistance in *An. culicifacies* indicated that DDT and malathion resistance did not reverse completely even after long‐term withdrawal of DDT (> 30 years) and malathion (9 years) from IRS, while a complete reversal of deltamethrin resistance was observed within 2–3 years of the withdrawal of deltamethrin IRS [59]. Furthermore, the reversion of resistance depends on intrinsic fitness ratios of homozygotes and heterozygotes and the frequency of the resistance gene and the nature of inheritance of the gene [59].

The mechanisms of insecticide resistance can broadly be classified into two types, i.e., target site resistance and metabolic resistance. Target site resistance refers to alteration of the insecticide binding site, rendering the insecticide ineffective. It is further subdivided into four major types: (1) Nicotinic acetylcholine receptor (nAChRs); (2) modified acetylcholine esterase (MACE)‐based resistance, where insects produce an altered acetylcholine esterase enzyme that leads to insensitivity to carbamates and organophosphates; (3) knockdown resistance (*kdr*), where mutations in voltage‐gated sodium channels (VGSCs), such as L1014F, cause resistance to pyrethroids and DDT; and (4) duplication of resistance to dieldrin (*Rdl*), where mutations in the GABA (gamma‐aminobutyric acid) receptor confer resistance to cyclodiene insecticides. Metabolic resistance, on the other hand, involves the enhanced detoxification of insecticides through the overexpression of enzymes such as esterases, cytochrome P450 monooxygenases, and glutathione S‐transferases [54]. The overproduction of cytochrome P450 enzymes plays a critical role in resistance to neonicotinoids [60]. Studies on resistance mechanisms have indicated the involvement of carboxylesterases for malathion resistance [61], glutathione S‐transferase (GST) for DDT resistance [30], and monooxygenases for pyrethroid resistance [62]. Few molecular studies on deltamethrin resistance have shown involvement of L1014F mutation for pyrethroid resistance and L1014S for DDT resistance, and the frequency of the *kdr* gene is very low and mostly heterozygous [49, 63]. Thus, studies so far have indicated the noninvolvement of the VGSC gene in conferring pyrethroid resistance in Indian *An. culicifacies* and the major mechanism of resistance being metabolic resistance [49]. Hence, the use of coformulated piperonyl butoxide–pyrethroid ITNs can be a viable option for pyrethroid resistance management in *An. culicifacies*. Notably, a study on deltamethrin‐resistant *An. culicifacies* in Chhattisgarh, India, showed the involvement of monooxygenases as a major mechanism associated with esterases and synergized by specific esterase synergists [62]. Hence, the use of coformulated piperonyl butoxide–pyrethroid ITNs (insecticide treated nets) can be a viable option for pyrethroid–resistance management in *An. culicifacies*.

Another aspect of relevance for control is the existence of this species as a species complex of five species designated as A, B, C, D, and E, which exhibit variations in various biological aspects such as prevalence, breeding habitat preferences, vectorial capacity, host feeding preferences, and insecticide susceptibility [5]. The data on different biological aspects of sibling species of different species could not be included in this review as they were not available retrospectively during the period of the review and also they were not a part of the experimentation of those investigations. Such strategies based on sibling species distribution are not being implemented in the country, as it is technically intensive and requires monitoring of sibling species among the field population. These varied biological attributes including insecticide resistance in vector species mainly of *An. culicifacies* species complex make the vector control complicated, mainly leading to a reduction in the efficacy of standard tools such as IRS and ITNs. Effective control demands species‐specific interventions based on the understanding of vector behavior and biology and innovative strategies tailored to address the control options suited for local ecological and insecticide resistance patterns.

For another important vector species, *An. fluviatilis*, data were available from 14 districts, which showed susceptibility to DDT, malathion, and deltamethrin with an exception for DDT in three districts (Angul, Kalahandi, and Mayurbhanj) reporting possible resistance. The data for this species also reported susceptibility to these three insecticides in an earlier reported review in 2017 [19]. Other two species *An. annularis* and *An. minimus* were reported mostly resistant to DDT, while limited data were available on malathion and deltamethrin, indicating that these species were completely susceptible, which is not in variance to the earlier reported review in 2017 [19].

In India, a strategy for the change of insecticides has been reactive [64], i.e., the successive replacement of an insecticide in use after its failure with a different class of insecticide led to the development of multiple resistance to different classes of insecticides due to the sequential selection. The insecticide resistance in malaria vectors is mainly due to the selection by insecticides in use in public health, but the possibility of selection due to the pesticide usage in agriculture cannot be ignored, as there are several such reports [65]. In India, the contribution of agricultural pesticide use in the development of insecticide resistance in malaria vectors exists [61] but could not be correlated mainly due to the lack of quality data to assess the impact.

The patterns of insecticide resistance as observed in Odisha, especially in *An. culicifacies* in the congruent districts of the three states, Andhra Pradesh, Chhattisgarh, and Telangana, were found to be triple resiatant to pyrethroids, DDT, and malathion [19]. *An. culicifacies* were triple resistant in the three congruent districts East Godavari, Srikakulam, and Visakhapatnam of Andhra Pradesh; Bastar, Dhamtari, Jashpur, Mahasamund, and Raigarh districts of Chhattisgarh; and Khammam district in Telangana. Later to the earlier review in 2017 [19], no data were available from the districts of the state’s congruent to Odisha.

The present scoping review collated the available data from the year 1993 and was the first dataset for consideration. Major limitation was the availability of insecticide resistance data with 28 records in the last > 30 years mostly on phenotypic resistance and very few on resistance mechanisms. Among them, most of the records are of *An. culicifacies*, while few records were for other important vectors. The low reporting of insecticide resistance data may also be due to the low density of mosquitoes in the field, leading to insufficient numbers of mosquitoes, i.e., at least 100 per insecticide, as per the protocol. Vector species other than *An. culicifacies* are reporting widespread insecticide susceptibility and few sporadic reports of resistance that are not of operational significance; however, this will not discount the need for more data. A lack of data on insecticide resistance and its mechanism/s is the major gap that influences the implementation of appropriate vector control strategies that are mostly chemical insecticide reliant, mainly synthetic pyrethroid interventions. The available data indicated a trend in the increase of pyrethroid resistance. The national strategy as of now is limited to the use of this insecticide, and a consideration of an alternate insecticide or strategy is needed. This will limit the useful life of synthetic pyrethroid in the future and is a caution to incorporate the use of insecticides with a novel mode of action from alternate insecticide classes such as neonicotinoids and pyrroles and interventions using insecticide–insecticide/synergist mixtures and coformulated products for vector control to manage vectors and insecticide resistance.

In addition, it is known that agriculture pesticides contribute to the selection of resistance in malaria vectors but yet not correlated with quantum of pesticide usage, which is important to strategize the insecticide resistance management tactics.

This review further indicated a lack of regular monitoring of insecticide resistance in the districts of the state, leading to gaps in the information in time and space, thus challenging the formulation of appropriate vector control strategies.

The National Centre for Vector Borne Disease Control (NCVBDC), India, has published the Operational Manual for Integrated Vector Management [66] and Framework for Malaria Elimination [11]. In Odisha, the vector control needs renewed strategies for the management of insecticide resistance, especially for *An. culicifacies*, which is the main vector of malaria and also multi‐insecticide‐resistant, including to pyrethroid. Presently, vector control interventions are reliant on pyrethroid IRS and LNs. Moreover, pyrethroid resistance is reported in this species in three endemic states, viz., Chhattisgarh, Madhya Pradesh, and Odisha, while in other states, reports on resistance are sporadic. The options available for the management of insecticide resistance include the introduction of novel insecticides/interventions and the use of coformulated products that have been evaluated in India and many other countries and are recommended by WHO for use, viz., IRS [clothianidin (neonicotinoid)]; insecticide mixtures [clothianidin + deltamethrin]; mixture LNs ‐insecticide +insecticide [chlorfenapyr (pyrrole) + alphacypermethrin]; or insecticide + synergist/insect growth regulator [alphacypermethrin/deltamethrin + piperonyl butoxide (synergist)/insect growth regulator] [54].

## 5. Conclusions

This scoping review provided a comprehensive assessment of insecticide resistance among *Anopheles* mosquito vector species in Odisha during the years 1993–2024 upto September 2025, which revealed an urgent need for regular monitoring of insecticide resistance and effective management of multiple insecticide‐resistant vectors. *An. culicifacies*, the primary malaria vector in the region, exhibits widespread resistance to multiple insecticides in use in public health, namely, DDT, malathion, and deltamethrin. This species showed triple resistance to insecticide in 10 southern districts, which is an impending threat for vector control, although for the present, intensity bioassays have indicated unlikely failure of ongoing pyrethroid interventions, but needs a caution to reduce selection pressure by implementing strategies for insecticide resistance management to increase useful life of existing insecticides. However, other species, such as *An. fluviatilis*, *An. annularis*, and *An. minimus*, were mostly susceptible, with sporadic reports of resistance, and are of low operational concern. The emergence of double and triple resistance in many districts revealed an urgent need for innovative and targeted interventions with new classes of insecticides with a novel mode of action and differently formulated insecticidal intervention products.

Furthermore, this scoping review identified several research gaps that need to be addressed. While the majority data were on *An. culicifacies*, data on other vectors such as *An. fluviatilis*, *An. annularis*, and *An. minimus* are limited, both on insecticide susceptibility and resistance mechanisms. Sibling species complexity in vectors remains understudied, complicating control strategies. The impact of agricultural pesticide use on resistance development in public health disease vectors remains unquantified.

## Funding

This study was supported by the Government of Odisha under Grant No. OURIIP‐21SF/ZO/80 to Kiran Bala Bhuyan. Tapan Kumar Barik was, in part, supported by ICMR under​ extramural grant 6/9‐7(314)/2023‐ECD‐II.

## Conflicts of Interest

The authors declare no conflicts of interest.

## Data Availability

The data that support the findings of this study are available from the corresponding author upon reasonable request.
